# Interstitial Lung Disease of the UIP Variant as the Only Presenting Symptom of Rheumatoid Arthritis

**DOI:** 10.1155/2015/205175

**Published:** 2015-06-07

**Authors:** Abhinav Agrawal, Braghadheeswar Thyagarajan, Sidney Ceniza, Syed Hasan Yusuf

**Affiliations:** Department of Internal Medicine, Monmouth Medical Center, Long Branch, NJ 07740, USA

## Abstract

Rheumatoid arthritis is a chronic inflammatory disease primarily manifesting with symptoms of joint pain. It also involves multiple organ systems in the body, including the lungs. Interstitial lung disease (ILD) is the most common form of pulmonary involvement in rheumatoid arthritis (RA). Without the typical symptoms such as chronic joint pain, establishing the diagnosis of RA could be quite challenging and a high index of suspicion is thereby required to diagnose ILD in patients with RA, thereby delaying treatment and increasing morbidity and mortality. We report a case of a 67-year-old Hispanic male with no previous history of rheumatoid arthritis or symptoms of typical joint pain who comes to the hospital only with the chief complaints of progressive worsening of shortness of breath for a duration of 6 months and was eventually diagnosed with ILD of the usual interstitial pneumonia variant with serologies positive for rheumatoid arthritis.

## 1. Introduction

Rheumatoid arthritis affects about 1% of the population [1]. Respiratory symptoms in rheumatoid arthritis can be due to a variety of conditions that affect the parenchyma, pleura, airways, or vasculature. The majority of respiratory manifestations occur within the first 5 years of disease [2]. Respiratory symptoms may precede onset of articular symptoms in 10–20% of cases [3]. Interstitial lung disease (ILD) is the primary pulmonary involvement in RA with prevalence ranging from 4% to 68% mostly in the age group of 50 to 60 years [4].

## 2. Case Presentation

Our patient is a 67-year-old Hispanic male who presented to our hospital for the chief complaint of progressive worsening of shortness of breath of 3 weeks duration and was admitted for acute respiratory distress due to interstitial lung disease of unknown etiology. His medical history includes coronary artery disease, status postpercutaneous coronary intervention in 2010, hypertension, hyperlipidemia, and diabetes mellitus type 2. He has a significant smoking history of 20 to 30 pack-years and a significant history of alcohol drinking consuming 12 cans of beer per day for 30 years. He has quit smoking and drinking for 10 to 15 years. He works as a janitor and has no significant occupational exposure to asbestos or silicone.

**Figure 1:**
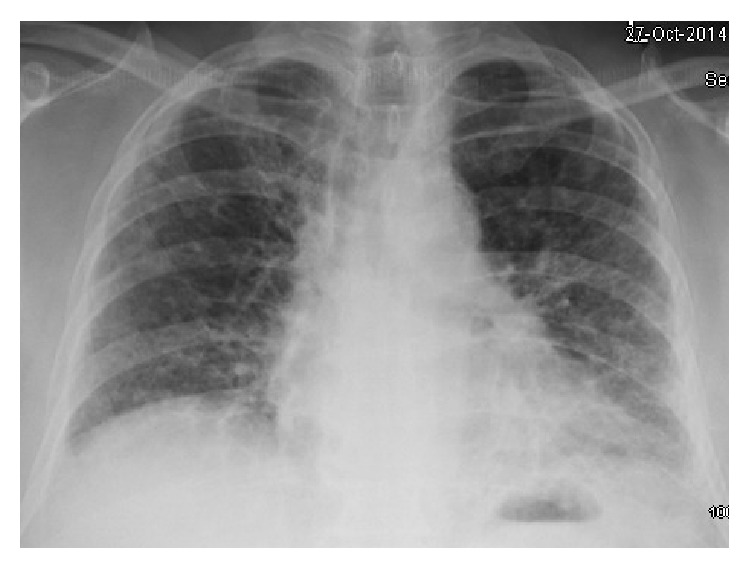
Chest X-ray taken 6 months prior to the day of admission showing mild hazy changes in bilateral lung fields.

Six months prior to the day of admission, the patient had complaints of dry cough for which he visited his primary care physician and was prescribed over-the-counter cough suppressants with no relief. The patient had a chest X-ray done at this time which showed mild hazy changes in bilateral lung fields (Figure 1). The patient continued to have dry cough and eventually developed progressive shortness of breath with no dyspnea at rest. The patient continued to ignore his symptoms until few weeks prior to the day of admission he had significant shortness of breath to the point to which he could not walk to the bathroom in his house. Concerned of this he came to the hospital for further evaluation.

In the ED, the patient was brought in by his daughter and he had significant shortness of breath during ambulation which caused him to rest after every few steps. His initial SpO2 on room air was 77% and he was put on 3-litre O2 on nasal canula and his SpO2 improved to 93%. His blood pressure was 134/76 mm of hg, heart rate was 78/min, and respiratory rate was 22/min. On physical examination the patient was awake, alert and oriented, his neck was supple with no jugular venous distension, his heart sounds were audible with no murmurs or gallops, and auscultation of his chest revealed bilateral breath sounds with significant velcro rales on all the lung fields bilaterally. Abdomen was soft and nontender and the patient had no focal neurological deficits. Examination of his extremities revealed no pedal edema but grade 3 clubbing of his finger nails. The patient denied fever, chills, hemoptysis, orthopnea or paroxysmal nocturnal dyspnea, recent travel, or sick contacts.

**Figure 2:**
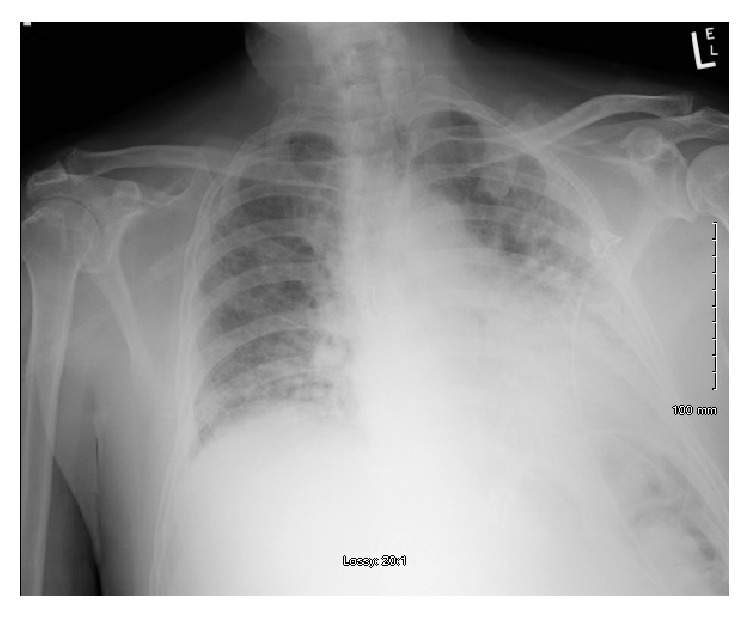
Chest X-ray showing worsening reticular and hazy markings throughout bilateral lung fields compared to the previous chest X-ray.

**Figure 3:**
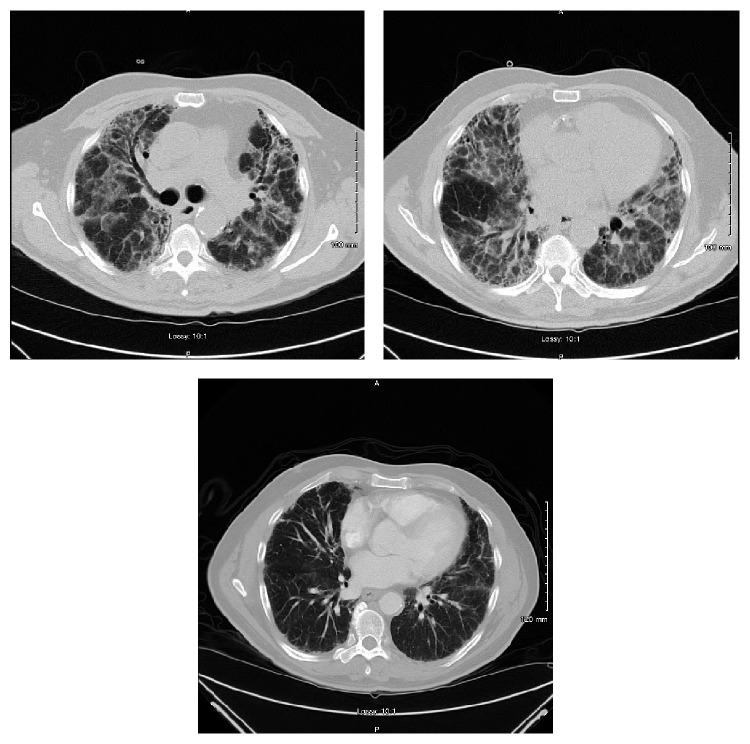
CT scan of the chest showing extensive honeycombing consistent with UIP.

**Figure 4:**
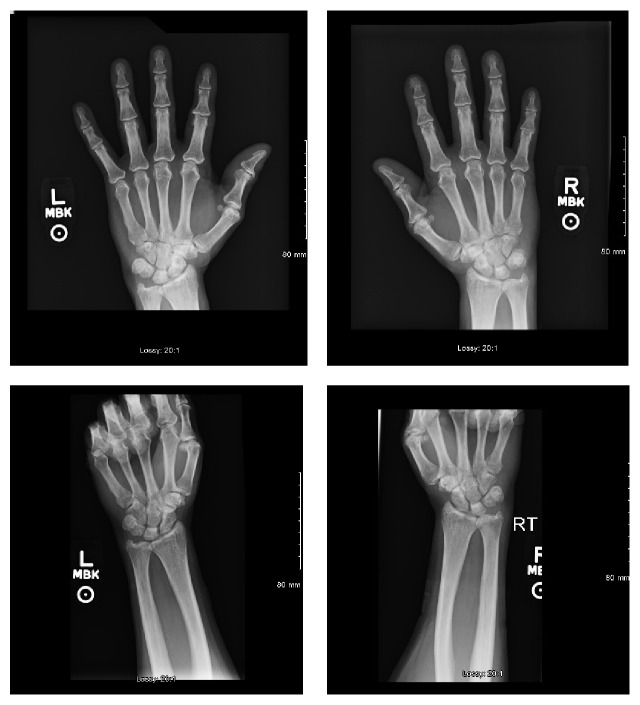
X-ray of the bilateral hands showing early inflammatory arthropathy.

Chest X-ray in the emergency department showed reticular and hazy markings throughout the both lungs, being worse compared to the previous chest X-ray (Figure 2). A CT scan of the chest showed extensive honeycombing and bronchiectasis of both lungs which were markedly worse when compared to a CT scan done 4 years ago (Figure 3). X-rays of both hands and wrists showed early inflammatory arthropathy but the patient denied any joint pain (Figure 4). 2D echocardiogram showed ejection fraction of 59% with mild mitral regurgitation and no pulmonary hypertension, which was not consistent with CHF.

Concerning his chest X-ray and CT scan findings, ILD was now the working diagnosis. The differential at this time was idiopathic versus rheumatoid arthritis. Laboratory data showed elevated erythrocyte sedimentation rate 98 (*N* 0–20), elevated C-reactive protein 17.4 (*N* ≤ 7.0), elevated rheumatoid factor 275 (*N* ≤ 10), and elevated cyclic citrullinated peptide >250 (*N* < 20). To exclude causes of falsely elevated rheumatoid factor, hepatitis C Ab was done which was negative. ANA and DsDNA were both negative.

The complete blood count, electrolytes, and renal and liver functions were within normal limits. Lung biopsy was avoided due to the complications of an invasive procedure.

A diagnosis of interstitial lung disease of the usual interstitial pneumonia (UIP) variant due to rheumatoid arthritis was made. The patient was given intravenous solumedrol 40 mg TID which was tapered and changed to oral prednisone 60 mg daily upon discharge. During his hospital stay he was on nasal O2 3 litres and had episodes of desaturation on ambulation; hence he was discharged with home oxygen. He was advised to continue the rest of his medications for his comorbidities and to follow up with his primary care physician and pulmonologist as outpatient. Eventually the patient was referred to a tertiary care center for lung transplant. The patient is currently on the waiting list for his lung transplant.

## 3. Discussion

Rheumatoid arthritis (RA) is a chronic and systemic inflammatory response, primarily affecting the joints [1]. Interstitial lung disease (ILD) is a well-established and debilitating extra-articular manifestation of RA with a median survival of 2.6 years versus 9.9 years of RA without ILD [5]. This is as a resultant of progressive pulmonary deterioration with lung fibrosis and RA's inherent complications. RA-ILD affects up to 20–30% of RA patients with a 2 : 1 predilection for males as compared to females [6].

RA-ILD, lacking its own agreed classification, is generally subdivided on the basis of its radiographic and histopathological identification, which correlates well with the 7 subtype seen in idiopathic interstitial pneumonia appearances. The usual interstitial pneumonia (UIP) histopathological pattern is more commonly seen in RA as compared to other connective tissue disorders (CTD) where nonspecific interstitial pneumonia (NSIP) is the more prevalent pattern [7]. The patient mentioned above has the UIP type of ILD. The UIP pattern of RA appears to predict poor outcome and apparent clinical deterioration (disease progression), in contrast to idiopathic UIP (a.k.a. idiopathic pulmonary fibrosis) where its lack of response to medical treatment is generally recognized. However data comparing the two groups is limited, revealing somewhat improved 5-year survival rate in RA-UIP as compared to IPF [8]. The other RA-ILD subtype, NSIP pattern, which usually is seen more in women and nonsmokers, seems to be more favorable in terms of survival and response to treatment based on limited studies [9].

Despite the lack of clarity in the pathogenesis of RA-ILD, several predisposing risk factors have been implicated in the possible development of ILD including male gender, smoking, older age, high RA disease activity, long duration of disease, genetic carriers of HLA-DRB1 ^*∗*^1502, HLA-B40, anti-trypsin, anti-CCP, and RF; however other studies reveal conflicting data on these associations [10–14]. It has been observed that there is a convincing association tobacco usage with the development of RA-ILD [15]. It was interesting to note that our patient was a long-standing smoker. Anti-CCP is highly specific for RA (about 98%) and multiple studies are showing it can appear prior to the development of arthritis [16]. An interesting cohort of patients is being increasingly recognized for the development of RA-ILD and positive anti-CCP serology w/o synovitis, which initially presented with dyspnea only like our patient. One theory for such subjects is that they belong to a pre-RA state, which could then accelerate to develop RA if genetically and environmentally susceptible. Thus, the clinical implication of positive CCP and RF serology in a patient with lung fibrosis but without RA, which we have described in our case report, remains even more so challenging.

To date, no cost-effective screening has been validated but a detailed history (including environmental exposure, pets) and physical exam remain the most valuable tool in the assessment of patients inflicted with this condition. PFTs are considered highly sensitive in particular, a reduced DLCO (diffusing capacity of the lung for carbon monoxide), seen even if other pulmonary function indices do no reveal restrictive abnormalities for instance in pseudo-normal PFTs created by combination of pulmonary fibrosis and coexisting emphysema [17]. Thus the specificity of DLCO is also reduced as emphysema can also affect the DLCO. The diagnosis and histopathological subtype of RA-ILD are best established with lung biopsy but due to its invasive risks, an acceptable and equally effective mode for diagnosing is utilizing HRCT where CT features, for instance, bilateral subpleural reticulation with or without honeycombing (UIP) and predominant ground glass appearance (NSIP), would be consistent with interstitial lung disease [18, 19] and a respectable 70% concordance with its histological diagnosis [20]. The CT chest of our patient had honeycombing (Figure 3) which was consistent with UIP pattern of ILD and an invasive procedure such as biopsy and its complications was avoided.

Treatment with anti-inflammatory and/or immunosuppressive agents is recommended regardless of the pattern of fibrosis [21]. Corticosteroids are the mainstay of therapy, particularly for cases of ILD where they may lead to regression on imaging and potential clinical improvement [22]. Similarly, our patient was treated with corticosteroids. Cyclophosphamide and azathioprine have been used with varying success [23]. Methotrexate, a first-line agent in the treatment of rheumatoid arthritis joint disease, is known to be associated with drug-induced pneumonitis, but fortunately this is rare. However, there is no evidence that this agent leads to progression of ILD [24]. There is considerable controversy as to whether antitumour necrosis factor (TNF) agents improve or worsen ILD. Studies evaluating this issue tend to be confounded by older age and prior use of methotrexate among participants. Similar controversy also exists for rituximab, with some studies reporting improvement [25] and other studies reporting development of ILD [26].

Adjuvant therapy for RA-ILD includes smoking cessation, management of gastroesophageal reflux disease, referral to pulmonary rehabilitation, supplemental oxygen, and vaccination against influenza and pneumococcal disease. All of this was implemented in the care of our patient. In the absence of active rheumatoid arthritis, patients with rheumatoid arthritis lung disease who fail to respond to therapy should be considered for lung transplant. In patients with a UIP pattern, work-up for transplant should be considered early. A retrospective review of Canadian patients with advanced lung disease found no difference in outcomes between patients with RA-ILD and those with IPF at 1 year following lung transplant, suggesting that transplant is a reasonable option for these patients [27]. Similarly our patient had absence of active RA and given the UIP pattern of ILD he was referred for lung transplant program.

## 4. Conclusion

The diagnosis of RA becomes quite challenging without the usual symptoms of joint pain. It is critical for a physician to assess the patient for systemic and articular signs and symptoms of connective tissue disease when evaluating a patient with pulmonary disease of unknown etiology as patients may initially present with pulmonary symptoms. Early diagnosis can lead to early initiation of treatment and early referral to lung transplant centers in qualifying patients, thereby decreasing morbidity and mortality.
